# Impact of health literacy, accessibility and coordination of care on patient’s satisfaction with primary care in Germany

**DOI:** 10.1186/s12875-015-0372-0

**Published:** 2015-10-22

**Authors:** Sibel Vildan Altin, Stephanie Stock

**Affiliations:** Institute for Health Economics and Clinical Epidemiology, University Hospital of Cologne, Gleuelerstr. 176-178, 50935 Cologne, Germany

**Keywords:** Health literacy, Quality of care, Primary care, Patient-reported outcomes

## Abstract

**Background:**

Although health policy makers call for the transformation of health care organizations to health literacy responsive ones, there is limited evidence on the care experiences of patients with limited health literacy skills (HL) in respect to health care quality. We explored if HL and patient-reported experiences regarding access to care and support in care-coordination in primary care organizations (PCO) have an impact on patients satisfaction with the care received by their personal general practitioner (GP).

**Methods:**

A nationwide representative survey was administered in a random sample of 1125 German adults. Binary logistic regression analyses were performed to determine whether HL and perceived access to and coordination of care were associated with satisfaction with care received in primary care adjusting for demographics and health status.

**Results:**

In the unadjusted as well as adjusted model, better accessibility of the primary care practice (*β*= 1.858; 2.032 *p* < 0.001) frequent support in care coordination by the general practitioner (*β* = 2.680; 2.820 *p* < 0.001) as well as sufficient HL (*β* = 0.888; 1.228 *p* < 0.05) were independent predictors of a higher satisfaction with care received in the general practice.

**Conclusion:**

German adults with sufficient HL and positive experiences regarding care coordination and access to care are more satisfied with care received by their personal general practitioner. This result is from major importance for primary care organizations intending to transform their processes and structures to respond to the health literacy needs of their patients more effectively.

## Background

The principles of equality and quality in health care provision are becoming the tipping points of health policy agendas worldwide [1, 2]. The efforts follow the main objective to improve the value of care by achieving better outcomes that matter to patients and reduce the costs required to deliver the outcomes [3]. According to this approach, high-quality care has to be needs-based and tailored to the risk profiles and respective health care needs of distinct patient populations. The attempt to thoroughly identify and respond to the health care needs of populations is expected to improve health care quality and equality and demands mutual transitions in health care governance and financing as well as service planning and delivery [4]. Additionally, it requires considerable investments in service delivery processes of health care organizations and an improvement of patient capabilities to navigate through the layers of health care systems that become more and more complex and demanding [5, 6]. Especially the latter aspects have been subject to intensive debates among scholars and health care decision makers in most developed countries recently [4, 7, 8]. Among others these debates originated from the growing evidence on the limited literacy skills in large parts of numerous populations to obtain, process, communicate, and understand basic health information and services as well as the undesirable outcomes these missing capabilities result in [9–11]. In this regard, various studies demonstrated that limited health literacy skills are associated with poor adherence to medication-regimes, insufficient self-management skills and more frequent hospitalizations and emergency care utilizations [12]. Other literature has emphasized that especially vulnerable groups such as chronically ill, deprived and elderly populations are affected by limited health literacy noting that these findings challenge the achievement of high health care quality as well as equality [13]. The overall recognition that differing health outcomes among diverse populations are also rooted in limited health literacy skills to use information for the own health lead to the overarching goal to respond to this issue on a system level [14]. In this regard, scholars and medical organizations such as the Institute of Medicine (IOM) recently proposed to transform healthcare organizations to health literacy responsive ones by redesigning their structures and processes to support low literate patients to navigate, understand, and use information and services to take care of their health [5, 7, 15, 16]. According to the approach of the IOM there are ten crucial attributes of health literate healthcare organizations (HLHO), that need to be considered when transforming an organization to a health literacy responsive one [7]. Among them, the integration of patient-centered care including the use of plain language by healthcare providers, the development and distribution of written/audio/visual health information tailored to the differing health literacy levels and needs of patients and the coordination of care are deemed most valuable to have an impact on patient outcomes [17, 18]. However, although these efforts seem very purposeful there is limited evidence on the actual interrelation of limited health literacy with patients care delivery experiences in general and quality of care in a narrower sense [19]. In this respect, there is much scientific work done on the impact of limited health literacy on health outcomes and health care utilization [12] but its interrelation with patient experiences with health care quality remains indefinite [20]. However, organizational change towards a more health literacy friendly environment requires a greater understanding of the factors affecting patient’s perception of the quality of healthcare delivery. In this regard, patient-reported access to care as well as experiences with care delivery (e.g. support in care-coordination) are common dimensions to determine health care performance and quality [21, 22] and are known to influence patients care experiences and satisfaction with care [23, 24]. Latter is also a frequently used measure to operationalize the overall care experiences of patients in a certain health care setting [25]. Subsequently, it would be worthwhile to investigate the interrelations between limited health literacy skills and patient’s experiences with care quality and their impact on the overall care experience of the patient, namely his satisfaction with the care received [19]. By examining these interrelations scholars would be able to identify if limited health literacy is an additional factor that influences the patients overall satisfaction with care delivery alongside already known quality of care factors such as care coordination and access to care. Such a finding could considerably support the argument to establish health literate health care organizations. By now, findings in this field are scarce and equivocal, especially with respect to primary care [24, 26]. However, clearer evidence is particularly relevant for primary care, which is regarded as a meaningful setting to diminish the literacy related inequalities in health care [27, 28].

In our study, we will fill this research gap by performing a nationwide representative survey among the German adult population to explore if health literacy skills and patient-reported experiences in regard to access to care and support in care-coordination in the primary care setting have an impact on patients overall satisfaction with the care received by their personal general practitioner (GP).

## Methods

### Study design and participants

The study involves computer-assisted telephone interviews with a nationally representative random sample of adults aged 18 and older living in Germany. Data was derived from the 2013 Commonwealth Fund International Health Policy Survey. The sample was contacted from February to May 2013 by random-digit dialing of both landlines and mobile phones covering whole Germany. Up to eight calls were made to establish contact. The responders were 11.0 %, defined as completed interviews (*N* = 1125) out of the overall sample members that could be contacted (*N* = 10.300). Since the survey was non-medical, there was no ethical approval required from the Ethical Review Board of the Medical Faculty of the University of Cologne, Germany. Participation in the survey was voluntary. Written informed consent was obtained from every participant before the questionnaire was answered. Confidentiality was maintained by data coding to eliminate the identification of data with personal information.

### Study variables

Patient demographic information included age, gender, educational attainment (low, middle, high educated), migration status and insurance type (public/private). Educational attainment was categorized according to the International Standard Classification of Education (ISCED) organizing educational attainment in three levels (low, middle and high education) [29]. Migration status was determined by country of birth. More specifically, respondents were classified as migrants when they were not born in Germany or born in Germany with at least one parent born in a foreign country.

Self-rated health status was assessed using one item asking “In general, how would you describe your own health?”. The item was answered on a five point Likert scale ranging from “poor” to “excellent”. In addition, a more objective indicator was added asking if someone was diagnosed suffering from a chronic condition such as diabetes, coronary artery disease, hypertension, asthma, or a depression. For the analysis self-rated health status (1 = fair/poor; 0 = excellent, very good, good) as well as was number of chronic conditions (1 = ≥2 chronic conditions, 0 = <2 chronic conditions) were analyzed as binary outcomes.

Health literacy was measured using a one-item screener retrieved from the Brief Health Literacy Screen (BHLS), a verbally administered self-report measure of functional health literacy. The screener item was developed by Chew and colleagues and has been validated against widely used measures of health literacy [30, 31] across a variety of settings [32–34]. The respondents were asked: “How often do you have problems learning about your medical conditions because of difficulty understanding written information?”. The item was answered on a five point Likert scale ranging from “always” to “never”. Following previous studies reporting the response option “sometimes” as a cut-point with highest sensitivity and specifity values to screen for limited health literacy, we coded respondents who reported to have rarely/never problems learning about their medical condition as having “no problem” and respondents who reported to have always/often problems as having a “problem” [33, 35]. “Don’t know” responses were considered as missing.

Perceived satisfaction with the care received by the general practitioner was measured using the item “How do you rate the overall medical care received in the last 12 months by your general practitioner?”. Response was assessed on a five point Likert scale ranging from “1 = poor” to “5 = excellent”. The item was analyzed as a binary outcome (1 = excellent, very good, good; 0 = fair, poor).

Perceived accessibility of the general practice was assessed using the item “When you call your regular doctor’s office with a medical concern during regular practice hours, how often do you get an answer that same day?”. Response was assessed on a five point Likert scale ranging from “always” to “never”. The item was analyzed as a binary outcome (1 = never/rarely/sometimes; 0 = always/often).

Perceived support in care coordination was measured using the item “How often does your regular doctor or someone in your doctor’s practice help coordinate or arrange the care you receive from other doctors and places?”. Response was assessed on a five point Likert scale ranging from “always” to “never”. The item was analyzed as a binary outcome (1 = never/rarely/sometimes; 0 = always/often).

### Statistical analysis

Demographic data was analyzed using means, frequencies, and cross tabulations to calculate descriptive statistics. Associations between the outcome (perceived satisfaction with care received by the general practitioner) and predictor variables (i.e. accessibility and coordination of care in primary care, health literacy) were examined by conducting bivariate analysis using chi-squared tests for independence. The main study hypothesis was examined by applying binary logistic regression analyses. The dichotomized item assessing the satisfaction with the care received by the general practitioner served as the dependent variable. We compared an unadjusted model with a sequential model that controlled for age, gender, educational attainment, migration status, self-rated health and number of chronic conditions. Missing values for a variable were not included in analysis using that variable. Data was analyzed with SPSS version 21. Statistical significance was assessed as *p* < 0.05.

## Results

### Participant characteristics

Table 1 displays the characteristics of our survey sample. Respondents are in average 52.4 (±17.73) years old, 60 % are female and 43.6 % have a high school education or less. Overall, 76.0 % of the sample has a good to very good health whereas 29.7 % is affected by two or more chronic conditions with hypertension and coronary artery disease being the most prevalent. Almost all respondents, (94.8 %) do have access to a general practice they consult on a regular basis.

**Table 1:** Demographic characteristics

Variable	N	%
Participants	1125	
*Age*		
Mean ± SD	52.43 ± 17.73	
Range	18 - 96	
18–34 years	227	20.2
35–49 years	262	23.3
50–64 years	330	29.3
≥ 65	306	27.2
*Gender*		
Female	680	60.4
Male	445	39.6
*Migration status*		
Non-migrant	921	81.9
Migrant	197	17.5
*Education degree*		
Middle school degree	192	17.1
Intermediate high school degree	298	26.5
Vocational degree	116	10.3
University entrance qualification	254	22.6
University degree	195	17.3
*Income*		
Below average	515	45.8
Average	191	17.0
Above average	220	19.6
Not sure	199	17.6
*Insurance status*		
Statutory health insurance	963	85.6
Private insurance	151	13.4
GP as regular doctor	1066	94.8
*Overall health status*		
Very good	408	36.3
Good	444	39,5
Fair	202	18.0
Poor	61	5.4
*Chronic conditions*		
< 2 chronic conditions	715	63.6
≥ 2 chronic conditions	334	29.7
Diabetes	99	8.8
Coronary artery disease	143	12.7
Hypertension	349	31.0
Asthma	114	10.1
Depression	134	11.9

Note: Percentages do not always add up to 100, due to missing values for several items: migration status (*n* = 7); education degree (*n* = 70); income (*n* = 199); insurance status (*n* = 11); health status (*n* = 10); chronic conditions (*n* = 76)

As presented in Table 2, in bivariate analysis, both the accessibility of the primary care practice as well perceived support in care coordination by the general practitioner were significantly related to satisfaction with care received by the GP in the last 12 months (*p* < 0.001). Sufficient health literacy skills were also related to reporting satisfaction with the care received by the GP (*p* < 0.05).

**Table 2:** Bivariate associations between patients health literacy skills as well as accessibility of care and care coordination experiences in primary care and satisfaction with care received in the last 12 month (*N* = 885)

Variable	Satisfaction with medical care received in the past 12 months at your regular doctor’s practice or clinic (excellent, very good, good)		
	*N*	(%)	*p*-value
*Accessibility of general practice*			
how often do you get an answer that same day?			<0.001
always/often	715	94.8	
never/rarely/sometimes	60	72.3	
*Assistance in care coordination*			
help coordinate or arrange the care you receive from other doctors and places?			<0.001
always/often	618	97.2	
never/rarely/sometimes	199	74.9	
*Health Literacy*			
problems when learning about medical conditions			<0.05
no problem (rarely/never)	589	93.5	
problem (always/often)	160	88.4	

### Association of health literacy, care coordination and accessibility with satisfaction received by the general practitioner

Table 3 summarizes the unadjusted and adjusted binary logistic regression models with satisfaction with primacy care as the dependent variable. In the unadjusted as well as adjusted model, better accessibility of the primary care practice, frequent support in care coordination by the general practitioner as well as sufficient health literacy skills were independent predictors of a higher satisfaction with care received in the general practice. Among the covariates, self-reported health status was associated with higher satisfaction with care (*p* < 0.01) received in the general practice.

**Table 3:** Unadjusted and adjusted regression models modeling the relationship between care accessibility, care coordination, health literacy and satisfaction with primary care

Variable	Satisfaction with medical care received in the past 12 months at your regular doctor’s practice or clinic (excellent, very good, good)			
	Model 1		Model 2	
	Beta (SE)	*p*-value	Beta (SE)	*p*-value
Accessibility	1.858 (0.424)	*p* < 0.001	2.032 (0.521)	*p* < 0.001
Assistance in care coordination	2.680 (0.455)	*p* < 0.001	2.820 (0.547)	*p* < 0.001
Health Literacy	0.888 (0.436)	*p* < 0.05	1.228 (0.536)	*p* < 0.05
Health status			1.242 (0.528)	*p* < 0.01
Total variance explained by the model (R^2^)	0.383		0.440	

Model 1: Unadjusted for socio-demographic variables

Model 2: Adjusted for education, gender, age, migration, self-reported health status, and number of chronic conditions

## Discussion

Although patient satisfaction with health care is recognized as an important dimension of health care quality [36] insights in the interrelations of patient’s health literacy and satisfaction with care are scarce [26]. Our findings of a representative nationwide survey of German adults indicate that health care provider related factors such as the accessibility of care and the provision of support in care coordination are both independently associated with patient’s perceived satisfaction with care received in the primary care practice. The presence of sufficient health literacy skills in terms of an individual’s ability to understand written information when learning about the own medical condition is also significantly associated with perceived satisfaction with primary care. These results indicate that apart from provider related factors in care delivery, the aspect of sufficient health literacy has also a considerable impact on the perception of quality of care in terms of satisfaction with health care delivery in the primary care setting.

Our findings support the notion that prompt access to care as well as comprehensive care coordination are key elements of high performing primary care organizations, subsequently having an impact on patient experiences with health care [37]. Prior studies examining the interrelations between care delivery in terms of coordinated and promptly accessible care and patient-reported experiences with care quality yield comparable findings in regard to satisfaction with care [24, 38].

While there has been extensive research on patient reported experiences on provider related determinants of care quality [39], patient related factors such as health literacy skills to deal with information on health care services effectively were less subject to investigations on perceived quality of care so far [5, 19]. Therefore, there is a paucity of evidence on the interrelations of patient’s health literacy skills and patient-reported outcomes on health care quality raising the necessity for novel frameworks and measurement instruments integrating the aspect of health literacy in quality of care frameworks [14]. In this regard, our research adds weight to the body of evidence on the influence of timely care management and access to care on patient satisfaction with care received and enlarges the insights into the impact of patient related factors on patient experiences with care. The latter finding provides evidence beyond the existing insights into the impact of patient’s socioeconomic status on perceived quality of care [40]. Although one study notifies a direct effect of health literacy skills on satisfaction with care, there is no data on the interactional effect of health literacy alongside other care related factors available [26]. This finding is also true for patient-reported experiences with quality of care in general. So far, studies elucidating the role of health literacy in health care mainly focused on health outcomes and health care utilization [12].

Our finding that health literacy plays a significant role alongside provider related care factors has considerable implications for the future development of primary care organizations. Therefore, primary care organizations aiming to deliver care in a way that patient satisfaction is improved need to address the issue of limited health literacy by transforming there organizational processes and structures according to the respective needs of patients [5, 15] to increase the value of care for vulnerable populations [3]. In this regard, leading organizations in the field of health service research and health care equity propose to implement novel organizational care models and frameworks that include health literacy as a key element and have the objective to support patients to navigate, understand, and use health information and services [5, 7, 14]. According to Koh and colleagues such a “health literate care model” requires a system approach enabling organizations to establish health literacy responsiveness as organizational value infused to all aspects of planning and operations, including self-management support, delivery system design, shared decision-making support as well as clinical information systems to track and plan patient care [14]. The corresponding “health literate health care organization” framework of the American Institute of Medicine (IOM) echoes this call for action by advocating health care organizations to establish techniques deemed helpful to identify and support patients with particular health literacy needs [7]. The use of plain language and confirmation of understanding when communicating with the patient are two exemplary techniques deemed helpful by the IOM. Subsequently, our results support this call for action by highlighting the importance of a health literacy responsive organizational environment for the patient’s experiences with care delivery.

This national study has several strengths that should be mentioned. First, it includes representative data of the German adult population obtained by generating a random sample. Second, it uses survey items developed in a scientifically rigorous process. Additionally the measurement approach to assess the outcome variable by asking to “think of care received by the personal GP in the last 12 months” is deemed appropriate since nearly all respondents had a personal GP they consulted on a regular basis.

The study limitations include the use of self-reported data to assess health care quality. Although this approach is a valuable way to assess the quality of care, objective measures are preferable [41]. In addition, the response of 11.0 % is relatively low, suggesting a potential selection bias, whose direction is unknown since data on the characteristics of non-responders is not available. One possible reason might be the rapid response design of the survey with a field time of 8 weeks. It needs to be pointed out that interviewers called potential survey participants at least eight times if they did not receive a response. However, there are similarities to other study populations of representative national survey studies in regard to age, gender and health status conducted in Germany recently [42, 43]. Future research should deepen the insights into the additional effect of health literacy on patient reported outcomes of health care quality by identifying the underlying causal pathways linking health care delivery patterns and individual patient skills to navigate the health care system.

## Conclusion

German adults with sufficient health literacy skills and positive experiences regarding care coordination and access to care are more satisfied with care received by their personal general practitioner. This result is from major importance for primary care organizations intending to transform their processes and structures to respond to the health literacy needs of their patients more effectively. In addition, it highlights the need for the establishment of new care models and frameworks in primary care aliening the current approaches with novel techniques that address the health literacy needs of populations in need for care.

## Abbreviations

PCO: Primary care organizations
IOM: Institute of Medicine
GP: General practitioner
HLHO: Health literate healthcare organizations
BHLS: Brief Health Literacy Screen
ISCED: International Standard Classification of Education
